# Swim Training Modulates Skeletal Muscle Energy Metabolism, Oxidative Stress, and Mitochondrial Cholesterol Content in Amyotrophic Lateral Sclerosis Mice

**DOI:** 10.1155/2018/5940748

**Published:** 2018-04-11

**Authors:** Damian Jozef Flis, Katarzyna Dzik, Jan Jacek Kaczor, Malgorzata Halon-Golabek, Jedrzej Antosiewicz, Mariusz Roman Wieckowski, Wieslaw Ziolkowski

**Affiliations:** ^1^Department of Bioenergetics and Physiology of Exercise, Medical University of Gdansk, 80-211 Gdansk, Poland; ^2^Department of Bioenergetics and Nutrition, Gdansk University of Physical Education and Sport, 80-336 Gdansk, Poland; ^3^Department of Neurobiology of Muscle, Gdansk University of Physical Education and Sport, 80-336 Gdansk, Poland; ^4^Department of Physiotherapy, Medical University of Gdansk, 80-211 Gdansk, Poland; ^5^Department of Biochemistry, Gdansk University of Physical Education and Sport, 80-336 Gdansk, Poland; ^6^Department of Biochemistry, Nencki Institute of Experimental Biology, 02-093 Warsaw, Poland

## Abstract

Recently, in terms of amyotrophic lateral sclerosis (ALS), much attention has been paid to the cell structures formed by the mitochondria and the endoplasmic reticulum membranes (MAMs) that are involved in the regulation of Ca^2+^ signaling, mitochondrial bioenergetics, apoptosis, and oxidative stress. We assumed that remodeling of these structures via swim training may accompany the prolongation of the ALS lifespan. In the present study, we used transgenic mice with the G93A hmSOD1 gene mutation. We examined muscle energy metabolism, oxidative stress parameters, and markers of MAMs (Caveolin-1 protein level and cholesterol content in crude mitochondrial fraction) in groups of mice divided according to disease progression and training status. The progression of ALS was related to the lowering of Caveolin-1 protein levels and the accumulation of cholesterol in a crude mitochondrial fraction. These changes were associated with aerobic and anaerobic energy metabolism dysfunction and higher oxidative stress. Our data indicated that swim training prolonged the lifespan of ALS mice with accompanying changes in MAM components. Swim training also maintained mitochondrial function and lowered oxidative stress. These data suggest that modification of MAMs might play a crucial role in the exercise-induced deceleration of ALS development.

## 1. Introduction

Amyotrophic lateral sclerosis (ALS) is an incurable, chronic neurodegenerative disease characterized by selective death of motoneurons in the motor cortex, brainstem, and spinal cord that control any muscle action [1]. It is important to note that causes of neurodegeneration in ALS occur outside of the nervous system. Overexpression of SOD1G93A in skeletal muscle not only initiates motoneuron death [2] but also causes profound muscle atrophy [2, 3]. Additional support of the view that muscle plays a key role in ALS pathogenesis is provided by the observations that the destruction of neuromuscular junctions has been linked to oxidative stress induced by tissue-specific breakdown of muscle mitochondria [4]. Conversely, there are plenty of data showing positive effects of regular exercise on mitochondria structure and function. Thus, analysis of the changes in muscle, especially related to skeletal muscle mitochondria, from transgenic animals can help to better understand the pathomechanism of the disease. Mitochondrial abnormalities are common in many forms of ALS and include morphological and functional aberrations, such as disruption of Ca^2+^ homeostasis, transport and bioenergetics, ROS overproduction, perturbed fission-fusion dynamics, and cell death initiation [5, 6].

These mitochondria dysfunction and oxidative stress are associated with abnormalities in the cell structures formed by the mitochondria and the endoplasmic reticulum membranes, which are called MAMs or MERCs [7–11].

MAMs, which contain protein and lipid (especially cholesterol) components, are involved in the regulation of Ca^2+^ signaling, mitochondrial bioenergetics, and apoptosis [12, 13]. Sala-Vila et al. [35] have shown that genetic deficiency of the key regulator of cholesterol efflux, caveolin-1 (Cav-1), in hepatic MAMs led to reduced MAM physical extension and integrity as well as aberrant-free cholesterol accumulation. Furthermore, the reduction of cholesterol in MAMs significantly promotes an association between MAMs and mitochondria [12], and this positively influences mitochondrial bioenergetics and structure [14, 15].

Cholesterol accumulation in mitochondria induced by the lack of Cav-1 protein reduces the efficiency of respiratory chain and antioxidant defenses [16]. Therefore, the attention of different research teams has been focused on the role of oxidative stress and the role of mitochondria and MERCs/MAMs in the mechanisms responsible for the development of neurodegenerative diseases [8, 17–20].

However, there are some promising observations on animal models of ALS which have shown that the lifespan can be prolonged. For example, swim training has been demonstrated to increase the lifespan of transgenic mouse [21]. Biochemical and physiological changes induced by exercise are quite well defined. Recent data has shown that swimming exercise modifies mitochondria structure and function due to changes in the concentration of mitochondrial cholesterol [15, 22].

To the best of our knowledge, there is no study explaining the mechanisms responsible for the induction of protective changes in skeletal muscle mitochondria of ALS mice induced by swim training. Therefore, the aim of this study was to investigate the effects of swim training and ALS progression on mitochondrial bioenergetics, oxidative stress, and changes in cholesterol concentrations in crude mitochondria isolated from the skeletal muscles of SOD1^G93A^ mice.

## 2. Materials and Methods

### 2.1. Animals

All experimental procedures were performed in accordance with European animal research laws (European Communities Council Directive 2010/63/EU). The experiments with animals were approved by the Local Ethics Committee (resolution number 11/2013) and the Polish Ministry of the Environment (decision number 155/2012).

Transgenic male B6SJL-Tg (SOD1^G93A^) 1Gur/J mice with the G93A human SOD1 mutation (ALS mice) (*n* = 36) and wild-type male B6SJL mice (*n* = 24) serving as controls for this mutant strain were purchased from The Jackson Laboratory (Bar Harbor, ME, USA).

The mice were housed in an environmentally controlled room (23 ± 1°C with a 12 h light-dark cycle); the mice received standard mouse chow and water ad libitum. After 14 days of acclimatization, the mice were randomly divided into the following groups according to disease progression and training status: ALS 0, ALS untrained mice with no visible signs of the disease (*n* = 8); ALS TER, ALS untrained mice (*n* = 8); and ALS SWIM, ALS trained mice (*n* = 8). Corresponding groups of wild-type (WT) untrained and trained mice were created: WT 0, WT untrained mice (*n* = 8); WT TER, WT untrained mice (*n* = 8); and WT SWIM, WT trained mice (*n* = 8).

To determine whether swimming training prolonged the lifespan of ALS mice, the following survival groups of mice were included: ALS S (*n* = 6) and ALS SWIM S (*n* = 6).

The mice were euthanized by cervical dislocation. The mice from the ALS 0 and WT 0 groups were euthanized on the 70th day of life. The mice from the ALS TER group were euthanized at the terminal stage of the disease (i.e., functional paralysis in both hind legs). The ALS SWIM, WT TER, and WT SWIM mice were euthanized at the same age as the ALS TER mice. The ALS S and ALS SWIM S (survival study) mice lived until there were signs of death.

### 2.2. Swim Training Protocol

Starting at 10 wks of age, the transgenic (groups ALS SWIM and ALS SWIM S) and control mice (group WT SWIM) underwent a training procedure according to the methods of Deforges et al. [21] with slight modification. Training was performed 5 times per week, and on each training day, the mice swam 30 min in 30°C water in a swimming pool with an adjustable flow (max rate of 5 l/min). On the 105th day of life, the frequency of training for the mice was reduced to 3 times per week, and the daily swimming time (max 30 min) and water flow (max 5 l/min) were set individually according to the abilities of the mice in the ALS groups. The training was performed until their 115th day of life for both the ALS and WT swimming mice.

### 2.3. Assessment of the ALS Mice Clinical Score

Starting at 8 wks, assessment of the clinical score of the ALS mice was performed according to [23].

### 2.4. Isolation of Skeletal Muscle Mitochondria

The skeletal muscle mitochondria were isolated, as previously described by Makinen and Lee [24] with slight modifications. The thigh muscles were rapidly removed, trimmed of visible connective tissue, weighed, and placed in 10 ml of ice-cold mitochondrial isolation buffer A (100 mM KCl, 50 mM Tris base, 5 mM MgCl_2_, 5 mM EDTA, and pH 7.4). The muscles were minced with scissors and incubated for 1 min with protease (10 ml of isolation buffer per 1 g of tissue, supplemented with protease (0.2 mg/ml)). After 1 min of incubation, the same volume of buffer A was added and homogenized using a Teflon pestle homogenizer. The homogenate was centrifuged at 700 ×g for 10 min. The supernatant was decanted and centrifuged at 4000 ×g for 10 min. The mitochondrial pellet was resuspended in 30 ml of suspension buffer B (100 mM KCl, 50 mM Tris base, 1 mM MgCl_2_, 1 mM EDTA, and pH 7.4, supplemented with 0.5% BSA) and centrifuged at 10000 ×g for 10 min. After this washing step had been repeated twice, the final mitochondrial pellet was resuspended in buffer MRB (250 mM mannitol, 5 mM HEPES, 0.5 mM EGTA, and pH 7.4) (skeletal muscle mass (mg) × 0.2 ml). All the steps were performed at 4°C.

### 2.5. Estimation of Cholesterol Content

The cholesterol content was measured in samples that had been normalized to the mitochondrial protein concentration per gram of tissue. The total lipids were prepared by vortexing 0.5 mg of sample with 1 ml of extraction medium (methanol/chloroform: 11 : 7). The mixture was then centrifuged at 15000 ×g for 10 min. The liquid (organic phase) was then transferred to a glass tube. The solvent was completely evaporated by drying at 80°C, and the pellet was later dissolved in 200 *μ*l of 1X Assay Diluent with vortexing until the solution was homogenous. The total cholesterol content was determined using a fluorometric assay kit (STA-390 Total Cholesterol Assay Kit, Cell Biolabs Inc., San Diego, CA, USA).

### 2.6. Determination of the Cav-1 Protein Level

The concentration of Cav-1 was measured using a Cloud-Clone Corporation (Katy, TX, USA) Caveoline-1 ELISA Kit (cat. number SEA214Mu) according to the manufacturer's instructions.

### 2.7. High-Resolution Respirometry

Respiration was measured at 37°C in a high-resolution respirometer (Oroboros, Oxygraph; Innsbruck, Austria) according to [25]. The respiration medium consisted of 110 mM sucrose, 60 mM K-lactobionate, 0.5 mM EGTA, 0.1% BSA essentially fatty acid free, 3 mM MgCl_2_, 20 mM taurine, 10 mM KH_2_PO_4_, and 20 mM K-HEPES, with pH 7.1. The software DatLab (Oroboros) was used for data analysis.

Mitochondrial respiration protocol: freshly isolated skeletal muscle mitochondria (0.1 mg of mitochondrial protein) were used for respiration measurement. In order to avoid any potential oxygen diffusion limitation, all the experiments were conducted after hyperoxygenation (450 nmol O_2_/ml). State 2 respiration was assessed with 2 mM malate, a complex I substrate. State 3 respiration was reached with 5 mM ADP and subsequently, 10 mM glutamate, another complex I substrate. 10 *μ*M cytochrome c was added to control for outer mitochondrial membrane integrity.

The respiratory capacity rate (RCR) was calculated as a ratio of state 3 to state 2 respiration, according to [26].

### 2.8. Measurement of Cytochrome c Oxidase, Citrate Synthase, Lactate Dehydrogenase, Creatine Kinase, and Catalase Activities

All enzyme activities were measured spectrophotometrically (Cecil CE9200, Cecil Instruments Limited, Cambridge, UK) in thigh muscle homogenates. The enzyme activities (except creatine kinase and catalase) were expressed as *μ*mol/minute/g of tissue. The cytochrome c oxidase (COX) activity was measured at 37°C according to [27]. The citrate synthase (CS) activity was measured at 37°C according to [28]. The lactate dehydrogenase (LDH) activity was measured at 30°C according to [29]. The two concentrations of pyruvate (PYR) were used to determine the maximal activity of lactate dehydrogenase characteristics for subunit M_4_ (LDH 2.1) in the presence of 2.1 mM PYR and subunit H_4_ (LDH 0.3) in the presence of 0.3 mM PYR. The creatine kinase (CK) activity was measured at 37°C using a RANDOX CK-NAC assay (cat. number CK522, Randox Laboratories Ltd., Crumlin, County Antrim, UK) according to the manufacturer's instructions. The CK activity was expressed as U/g of tissue. The catalase (CAT) activity was measured at 30°C according to [30]. The CAT activity was expressed as nmol/min/g of tissue.

### 2.9. Proteomic Analysis

The level of investigated proteins was estimated with the use of liquid chromatography-MS3 spectrometry (LC-MS/MS), and the LC-MS/MS was conducted by the Thermo Fisher Center for Multiplexed Proteomics (Dept. of Cell Biology, Harvard Medical School, Cambridge, MA, USA). The samples were prepared as previously described [31] with some modifications. Peptide fractions were analyzed using an LC-MS3 data collection strategy on an Orbitrap Fusion mass spectrometer (Thermo Fisher Scientific Inc., Waltham, MA, USA). To visualize the similarities and differences between the studied groups, principal component analysis (PCA) was performed using the “R” program.

### 2.10. Manifestation of Oxidative Stress

The thiol groups (SH) [32] and lipid dienes [33] were measured in the skeletal muscle homogenates. In addition, lipid dienes were also assessed in crude mitochondria. The values of the thiol groups were expressed as mmol/g of tissue, and the lipid dienes were expressed as an oxidation index 233/215.

### 2.11. Data Analysis

Statistical analyses were performed using a software package (Statistica v. 13.0, StatSoft Inc., Tulsa, OK, USA). The results are expressed as the mean ± standard error (SE). The differences between the means were tested using two-way ANOVA. If a difference was detected in the ANOVA model, the significant differences were determined using Tukey's post hoc test. To verify the significance of the small, swim training-associated changes (ALS TER versus ALS SWIM), an LSD post hoc test or Student's *t*-test was used. For survival assessment, Kaplan–Meier survival curves and a log-rank test were performed. The results were statistically significant at *p* < 0.05.

## 3. Results

### 3.1. Beneficial Effects of Swim Training in the ALS Model

To assess the effects of swim training on the survival of ALS mice, we subjected the animal group to a regimen of regular exercise (as described in the Materials and Methods). The exercise protocol started at the presymptomatic stage (70 days of age) and ended when the mice were 115 days of age. Median survivals were 126 days of age in the sedentary mice and 135.5 days of age in the swimming group of ALS mice (*p* = 0.019) (Figure S1A). On the day of euthanasia, the mice in the ALS 0, ALS TER, and ALS SWIM groups had clinical scores of 0, 4.5 ± 0.2, and 2.8 ± 0.7 points, respectively.

Over the course of the disease, all the ALS mice lost weight as a result of progression of the disease. We therefore monitored body weight changes in all the groups (sedentary and exercise) from the beginning of the training protocol until death (Figure S1B).

### 3.2. The Effect of Development of ALS Disease and Swim Training on Cav-1 Protein Level and Cholesterol Content in Skeletal Muscle Tissue and Mitochondria

**Figure 1:**
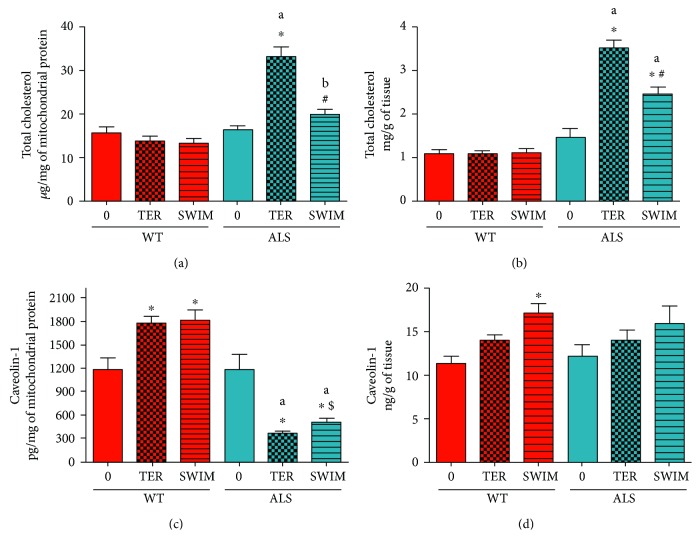
Cholesterol content and the Caveolin-1 protein level in skeletal muscle crude mitochondria and homogenates in the ALS and WT mice. Cholesterol content was assessed in skeletal muscle crude mitochondrial fraction (a) and homogenates (b). The Caveolin-1 protein level was measured in skeletal muscle crude mitochondrial fraction (c) and homogenates (d). There were significant differences between the groups: ^∗^ *p* < 0.05 versus ALS 0 or WT 0, respectively, ^#^ *p* < 0.05 versus ALS TER, ^$^ *p* < 0.05 versus ALS TER (unpaired Student's *t*-test); ^a^ *p* = 0.0001 and ^b^ *p* = 0.02 between the indicated ALS and corresponding WT groups. The data are presented as the means ± SEM (*n* = 8 in each group).

The differences in lifespan and clinical score at the day of euthanasia between the groups were accompanied by changes at cellular and mitochondrial levels due to the swim training. Therefore, we measured the level of cholesterol content and the level of Cav-1 protein, which is a marker of contact sites between the mitochondria and ER. At the terminal stage of the disease (ALS TER), the cholesterol content of the crude mitochondrial fraction doubled versus ALS 0 (*p* = 0.0001). Furthermore, swim training decreased the accumulation of cholesterol in the crude mitochondrial fraction (ALS TER versus ALS SWIM, *p* = 0.0001) (Figure 1(a)).

During progression of ALS, accumulation of cholesterol in the skeletal muscle homogenate was observed (*p* = 0.0001). Swim training induced a partial reduction in cholesterol accumulation (ALS SWIM versus ALS 0 and ALS TER, *p* = 0.0002) (Figure 1(b)).

There were no changes in the cholesterol content in the crude mitochondrial fraction and skeletal muscle homogenates of the WT animals (Figures 1(a) and 1(b)).

Cholesterol content in the crude mitochondrial fraction correlated to the Cav-1 protein level (*r* = −0.65, *p* = 0.0001; the Pearson product-moment correlation). At the terminal stage of disease (ALS TER), the Cav-1 level in the crude mitochondrial fraction decreased dramatically to approximately 30% of ALS 0 (*p* = 0.0003) (Figure 1(c)). In the ALS SWIM group of mice, a 35% higher Cav-1 level than in the ALS TER group was observed (*p* = 0.0242, unpaired Student's *t*-test; Figure 1(c)). There was an increase in the Cav-1 level for the crude mitochondrial fraction in both the WT TER and WT SWIM groups (*p* = 0.0114 and *p* = 0.0047, resp.) compared to the WT 0 group (Figure 1(c)).

During the progression of ALS, there was no significant change in the Cav-1 protein level in the skeletal muscle homogenate (Figure 1(d)). No changes in the Cav-1 level were observed in the WT 0 and WT TER groups, but a higher level of Cav-1 protein in the WT SWIM group was detected versus WT 0 (*p* = 0.0228) (Figure 1(d)).

### 3.3. The Effect of the Development of ALS Disease and Swim Training on Skeletal Muscle Energy Metabolism Systems

Changes in the Cav-1 level and cholesterol content in the crude mitochondrial fraction (MAMs/MERCs) may influence mitochondrial bioenergetics. Because of this possibility, we checked whether ALS progression and/or swim training may change activities of mitochondrial enzymes and the mitochondrial RCR.

**Figure 2:**
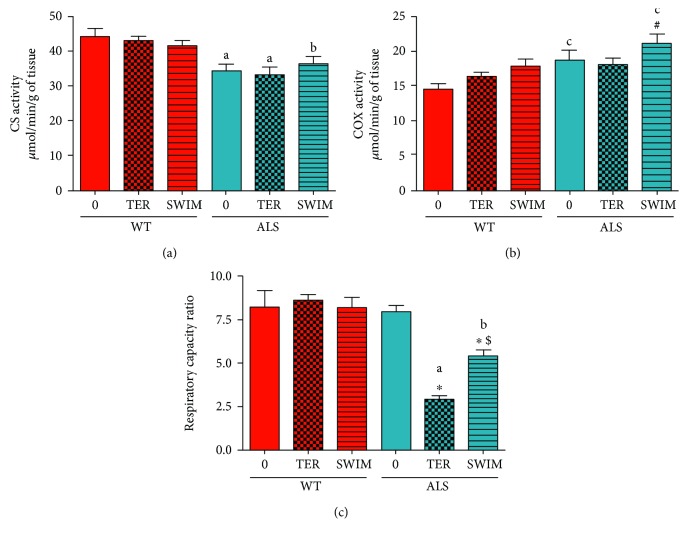
Citrate synthase, cytochrome c oxidase activities, and the respiratory capacity rate in skeletal muscle of ALS and WT mice. Citrate synthase (CS) (a) and cytochrome c oxidase (COX) (b) were measured in skeletal muscle homogenates. The RCR was assessed in skeletal muscle crude mitochondria (c). There were significant differences between the groups: ^∗^ *p* < 0.05 versus ALS 0, ^#^ *p* < 0.05 versus ALS TER, ^$^ *p* < 0.05 versus ALS TER (LSD post hoc test); ^a^ *p* < 0.0002, ^b^ *p* < 0.01, ^c^ *p* < 0.05 between the indicated ALS and corresponding WT groups. The data are presented as the means ± SEM (*n* = 8 in each group).

The activity of CS was reduced in all the ALS groups of mice compared with the corresponding WT mice (Figure 2(a)).

The COX activity increased in the ALS SWIM versus the ALS TER groups (*p* = 0.0483) as well as the corresponding WT group of mice (*p* = 0.0385). No changes in these enzyme activities were observed in the WT groups (Figure 2(b)). There was an almost 65% reduction in the RCR parameter in ALS TER versus ALS 0 (*p* = 0.0001). Training improved RCR in ALS SWIM compared to ALS TER mice (*p* = 0.0067, LSD post hoc test) (Figure 2(c)). There were no significant changes in RCR for any WT group of mice (Figure 2(c)).

The reduction of RCR and CS activity led us to determine whether ALS progression and/or training might have an influence on glycolysis and the creatine kinase system of ATP resynthesis.

**Figure 3:**
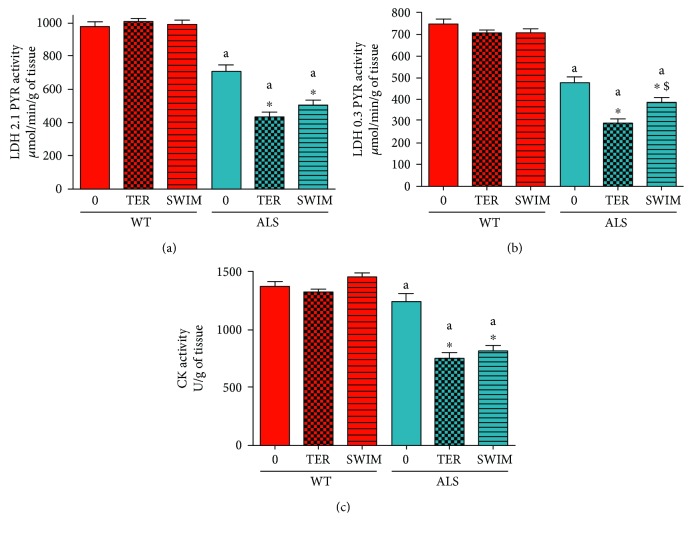
Lactate dehydrogenases and creatine kinase activities in skeletal muscle of ALS and WT mice. Lactate dehydrogenases with 2.1 mM pyruvate (LDH 2.1) (a), 0.3 mM pyruvate (LDH 0.3) (b), and creatine kinase (CK) (c) were measured in skeletal muscle homogenates. There were significant differences between the groups: ^∗^ *p* < 0.05 versus ALS 0, ^$^ *p* < 0.05 versus ALS TER (LSD post hoc test); ^a^ *p* < 0.0002, ^b^ *p* < 0.05 between the indicated ALS and corresponding WT groups. The data are presented as the means ± SEM (*n* = 8 in each group).

The activity of LDH 2.1 was lower in all groups of ALS mice than in WT mice (*p* = 0.0001). There was also a reduction in LDH 2.1 activity in ALS TER (*p* = 0.0001) and ALS SWIM (*p* = 0.0003) versus ALS 0 (Figure 3(a)). Similar changes were observed in LDH 0.3 (*p* = 0.0001). However, there was a small, swim training-associated increase (29%) in LDH 0.3 activity (ALS SWIM versus ALS TER) (*p* = 0.0268, LSD post hoc test) (Figure 3(b)). No changes in these enzymes activities were observed in the WT groups of mice (Figures 3(a) and 3(b)). The activity of CK was lower in all groups of ALS mice than in WT mice. There was also a decrease in CK activity in the ALS TER and ALS SWIM versus ALS 0 (*p* = 0.0001) (Figure 3(c)).

**Figure 4:**
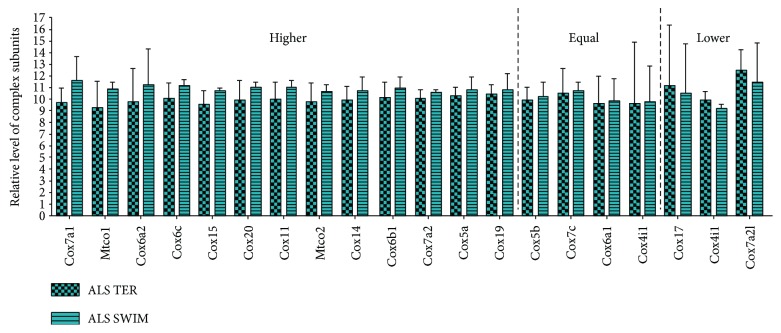
The relative level of complex IV subunits in skeletal muscle of the ALS 0, ALS TER, and ALS SWIM mice. The levels of 21 subunits that are part of complex IV of the mitochondrial respiratory chain in skeletal muscle for the ALS 0, ALS TER, and ALS SWIM groups. Subunits analyzed: Cox7a1, Mtco1, Cox6a2, Cox6c, Cox15, Cox20, Cox11, Mtco2, Cox14, Cox6b1, Cox7a2, Cox5a, Cox19, Cox5b, Cox7c, Cox6a1, Cox4i1, Cox17, Cox4i1, and Cox7a2l. The data are presented as the means ± SD (*n* = 3 in each group).

To determine whether the differences in the mitochondrial respiratory capacity rate and activity of mitochondrial enzymes between ALS 0, ALS TER, and ALS SWIM are related to the level of mitochondrial respiratory chain complexes, we investigated the level of individual subunits from OXPHOS. In our analysis, we took into consideration 100 identified subunits from the mitochondrial respiratory chain complexes and the ATP synthase complex. PCA enabled linear transformation of 100 variables (the levels of individual OXPHOS subunits) into a two-dimensional space but simultaneously retained the maximum amount of information about individual variables that were taken for the analysis. Created with the use of the new PCA variables PC1 and PC2, describe similarities and differences between the ALS 0, ALS TER, and ALS SWIM mouse groups (Figure S2A). Much better results are shown in graphic visualization (3D graph) of the results with the use of three newly created PCA variables (PC1, PC2, and PC3) from the 100 analyzed OXPHOS subunits (see Figure S2B) where it is clearly visible that the profile of the OXPHOS subunit level of ALS TER and ALS SWIM are practically identical and differ from the one created for the ALS 0 group. This difference means that swim training has no effect on the level of respiratory chain subunits in the ALS TER mice when they are analyzed globally, and this result cannot explain the improvement in the RCR parameter. Interestingly, we see differences between the ALS 0 and ALS TER groups that indicate that during disease development, the level of respiratory chain complexes is impaired, which can explain the attenuation of the OXPHOS function that is visible as RCR reduction. Interestingly, the level of most identified subunits of complex IV in the ALS SWIM mice is increased versus the ALS TER mice (Figure 4).

Similarly, we investigated the level of enzymes involved in the glycolysis process. As seen in Figure S2C, the profile of the levels of glycolytic enzymes in the ALS SWIM mice is more similar to that in the ALS 0 individuals.

### 3.4. The Effect of the Development of ALS Disease and Swim Training on Oxidative Stress

**Figure 5:**
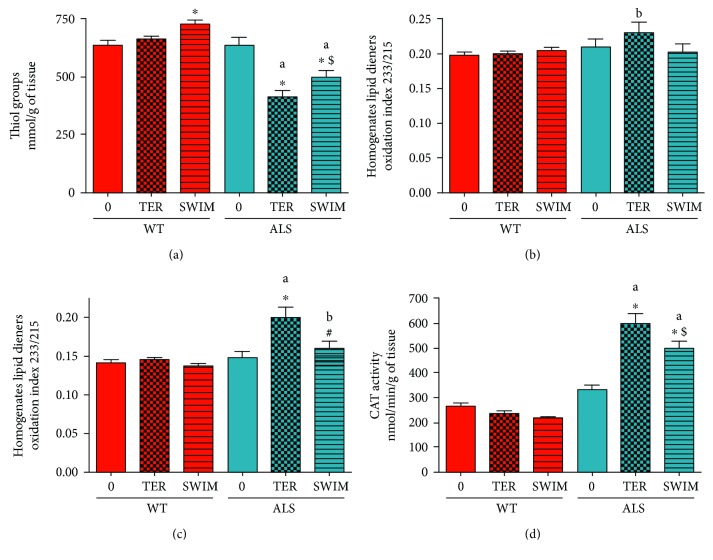
Oxidative stress parameters (thiol groups and lipid dienes) in skeletal muscle homogenates and crude mitochondria, and catalase activity in skeletal muscle of the ALS and WT mice. Thiol groups were measured in skeletal muscle homogenates (a). Lipid dienes were assessed in skeletal muscle homogenates (b) and the crude mitochondrial fraction (c). Catalase (CAT) activity was measured in skeletal muscle homogenates (d). There were significant differences between the groups: ^∗^ *p* < 0.05 versus ALS 0 or WT 0, respectively, ^#^ *p* < 0.05 versus ALS TER, ^$^ *p* < 0.05 versus ALS TER (LSD post hoc test); ^a^ *p* < 0.0002, ^b^ *p* < 0.01 between the indicated ALS and corresponding WT groups. The data are presented as the means ± SEM (*n* = 8 in each group).

Dysfunction of mitochondrial OXPHOS and the reduction of anaerobic energy production systems might induce oxidative stress. Therefore, we measured markers of macromolecule damage from free radicals in skeletal muscle homogenates and crude mitochondria fractions. At the terminal stage of the disease, we observed higher SH group oxidation in the ALS sedentary group of mice (ALS TER) than in the ALS 0 group of mice (*p* = 0.0001). A higher level of oxidative stress markers was also observed in the trained ALS mice (*p* = 0.0001). There is also a small, swim training-associated change in SH group oxidation (ALS TER versus ALS SWIM), (*p* = 0.007, LSD post hoc test) (Figure 5(a)). Changes that were similar (but not significant) were observed in lipid markers (Figure 5(b)). Swimming training in the WT mice resulted in a higher level of SH groups in skeletal muscle homogenates of the WT SWIM versus WT 0 groups (*p* = 0.0214). No changes in lipid peroxidation markers were observed in the WT mice homogenates (Figures 5(a) and 5(b)).

Additionally, we measured markers of lipid peroxidation in the crude mitochondrial fraction isolated from skeletal muscle. At the terminal stage of the disease, we observed higher lipid peroxidation in the crude mitochondrial fraction in the ALS sedentary group of mice than in the ALS 0 group (*p* = 0.0001). There was a reduction in lipid peroxidation in the mitochondria of the ALS SWIM versus ALS TER groups (*p* = 0.0163). No changes were observed in the WT groups of mice (Figure 5(c)).

Moreover, we measured activity of an antioxidant enzyme CAT. At the terminal stage of the disease, we observed higher CAT activity in the ALS sedentary group of mice (ALS TER) than in the ALS 0 group of mice (*p* = 0.0002). A higher level of CAT activity was also observed in the trained ALS mice (*p* = 0.005). There is also a small, swim training-associated change in CAT activity (ALS TER versus ALS SWIM), (*p* = 0.018, LSD post hoc test). No changes in CAT activities were observed in the WT mice (Figure 5(d)).

## 4. Discussion

In the present study, we demonstrated that the beneficial effects of forced swimming on the lifespan of ALS mice might be related to changes in skeletal muscle oxidative stress and bioenergetics. For the first time, we also showed that those protective changes induced by swim training were accompanied by lowering cholesterol content and raising the Cav-1 protein level in the skeletal muscle crude mitochondrial fraction. Due to the fact that mitochondrial cholesterol and Cav-1 are markers/components of mitochondrial and ER contact sites, we may also surmise that these changes are related to MAMs/MERCs modification in ALS mice skeletal muscle.

Our findings imply that the reduction in cholesterol contents, improvement in bioenergetics, and attenuation of oxidative stress may be a result of an increase in the Cav-1 protein level in the crude mitochondria of the skeletal muscle in trained ALS mice. As we previously indicated, these changes might be related to a dynamic physiological process that may help mitochondria adapt to stress conditions [15, 22, 34].

### 4.1. The Effects of Development of ALS Disease and Swim Training on the Cav-1 Protein Level and Cholesterol Content in Skeletal Muscle Tissue and Mitochondria

To the best of our knowledge, there are no data showing changes in cholesterol content and the Cav-1 protein level in skeletal muscle crude mitochondria of trained ALS mice and ALS mice at the terminal stage of the disease. However, Cutler et al. [58] showed that there is an increase in ceramides and cholesterol esters in the spinal cord of ALS patients and transgenic mice. Our data demonstrate reduced Cav-1 protein level in crude mitochondria and accumulated cholesterol content in skeletal muscle homogenates and crude mitochondrial fraction during the course of disease. Interestingly, swim training might reduce the accumulation of cholesterol in skeletal muscle and even maintain the cholesterol content in crude mitochondria close to the level observed before the onset of disease symptoms. These changes seem to be related to the Cav-1 level. The Cav-1 membrane scaffolding protein is enriched in MAM domains and plays a central role in the recruitment and regulation of cholesterol and steroid levels in these membrane regions [35]. The lack of Cav-1 is related to free cholesterol accumulation in mitochondrial membranes, increased membrane condensation, and reduced efficiency of the respiratory chain and intrinsic antioxidant defenses [16]. Moreover, changes in the Cav-1 protein and lipid composition in the mitochondria may result in significant reduction in the extension and/or stability of ER-mitochondria contact sites [35]. Recent reports showed that mutant SOD1 prevented ER membranes from binding to mitochondria [8]. According to these findings, we suspect that the Cav-1 protein may act as a critical regulator of MAM functionality in the presence of mutant SOD1 [8, 36]. The collapse of MAMs in ALS was previously observed (for review, see [7]). We assume that the reduction of contact sites between the ER and mitochondria that induces mitochondrial dysfunction and progression of ALS may also be related to changes in mitochondrial Cav-1 and cholesterol content. Our suppositions are based on the following assumptions. First, the Cav-1 protein plays a critical role as a modifier of mitochondrial respiratory chain function, the antioxidant enzyme defense system, and mitochondrial biogenesis under oxidative stress conditions [37]. Second, the increase in the Cav-1 protein that resulted in lowering the cholesterol content in the crude mitochondrial fraction seems to play a main role in exercise-induced mitoprotection via remodeling of MAMs [15, 22].

### 4.2. The Effects of ALS Disease and Swim Training on Skeletal Muscle Energy Metabolism Systems

#### 4.2.1. Mitochondrial Energy Metabolism in the Skeletal Muscle of the ALS Mice

In this study, we showed that CS activity, which is a marker of mitochondrial content [38], decreased not only at the terminal stage but also at the presymptomatic stage of the disease. Recently, it was reported that the activity of oxoglutarate and succinate dehydrogenases were reduced in the gastrocnemius muscle of SOD1^G93A^ mice [39]. Therefore, we may postulate that ALS is related not only to mitochondrial dysfunction but also to mitochondrial loss in skeletal muscle, as previously observed in the ALS spinal cord [40]. We observed a trend of higher CS activity in the ALS SWIM group of mice. This tendency may be the result of training-induced inhibition of the reduction in the number of mitochondria.

Interestingly, the COX activity was similar between the WT and ALS groups of mice. We also found higher activity for this enzyme in the ALS SWIM versus ALS TER groups, which is considered to be a marker of respiratory chain function improvement induced by training. In ALS patients, reduced COX activity in spinal cords was observed [40]. However, unchanged COX activity was also noted in the skeletal muscle [41, 42].

Although there was no change in COX activity between the ALS 0 and ALS TER groups, our data also demonstrate that during the course of the disease, lowering of the respiratory chain function was observed. Our data agree with previous observations in ALS skeletal muscles and the nervous system [43, 44]. Furthermore, reduced RCR at the terminal stage of disease implies that mitochondria have a low capacity for substrate oxidation and ATP turnover as well as a high proton leak, which is observed in dysfunctional mitochondria [45]. The improvement of RCR observed in dysfunctional mitochondria from the skeletal muscle of the trained ALS mice leads us to postulate that swim training may not only induce mitochondrial protection but also ameliorate mitochondrial function.

#### 4.2.2. Anaerobic Energy Metabolism in the Skeletal Muscle of ALS Mice

In this study, we found lower activity of CK and LDH in the skeletal muscle of the ALS mice than in that of the WT mice. Furthermore, the progression of the disease was related to a greater reduction in both LDH and CK activities.

Our data agree with previously published data showing lower activity of CK in the skeletal muscle of ALS mice [46]. Decreased LDH activity was also documented in a mouse model of motor neuron diseases and in dystrophic mice [47]. In the skeletal muscle of the SOD1^G86R^ mice, the activity of phosphofructokinase, the rate-limiting enzyme of glycolysis, was also reduced [48]. The lower LDH and CK activity in the skeletal muscle of ALS animals could also result from skeletal muscle membrane damage and leakage of these enzymes to the blood. A higher level of LDH and CK activity was present in the serum of ALS patients [49, 50]. We also found a trend indicating higher activity of LDH 0.3, which might be considered an indicator that confirms sustained oxidative metabolism in ALS muscles after swim training.

#### 4.3. The Effects of ALS Disease and Swim Training on Oxidative Stress

Notably, changes in energy production preceded not only the first symptoms of the disease but also the onset of skeletal muscle oxidative damage. Our data correlate with previous reports that suggest that metabolic defects in energy production pathways could contribute to disease progression in ALS [39].

It is likely that the lack of changes in oxidative stress markers before the onset of the disease indicate that lipid and protein peroxidation in skeletal muscle may be related to mitochondrial dysfunction and energy deficiency. These noxious changes in ALS pathology may be initiated via ROS generation, protein misplacement, or aggregations (for a review, see [51, 52]).

Our data demonstrate that ALS progression induced macromolecular damage of skeletal muscle through free radicals at the cellular and mitochondrial levels. Numerous studies have found evidence of increased oxidative stress in ALS pathogenesis. More oxidative damage was also observed in the skeletal muscle of ALS animal models [3, 53–55]. Some authors suggested that oxidative stress prevention is a key element in neuroprotection in ALS and other neuromuscular disorders [56, 57]. In this study, we also documented that swim training induced adaptive changes in the skeletal muscles of ALS and WT mice that resulted in decreased oxidative stress. One of the reasons oxidative stress is induced via mitochondrial dysfunction is the Cav-1-related increase in the cholesterol content in mitochondria [16]. Furthermore, an accumulation of cholesterol esters in the spinal cord of an ALS patient mediates oxidative stress-induced death of motor neurons [58].

Further studies are needed to investigate the mechanisms responsible for Cav-1-related MAM regulation. However, the use of isolated mitochondria in some cases, including ALS pathomechanism, may cause the lack of cellular context. Because of these limitations, additional studies with the use of cellular models of ALS can be useful to provide the direct evidence for the role of MAM modification in ALS. These studies could help to understand the swim-induced slowing down the development of the ALS disease, what would be of practical benefit.

## 5. Conclusions

**Figure 6:**
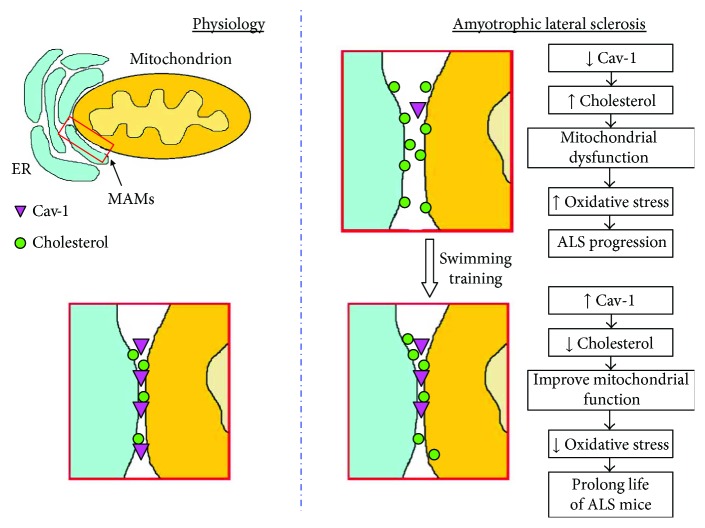
The Cav-1-dependent hypothesis of swim training-induced MAM modification leading to slowing down the development of the ALS disease. Under physiological conditions: appropriate levels of Cav-1 and cholesterol concentrations are presented in the skeletal muscle mitochondria. Progression of ALS is related to decreased Cav-1 level and increased cholesterol content in crude mitochondria, which leads to mitochondrial dysfunction, oxidative stress propagation, and ALS progression. Swim training induces the opposite effects, for example, increased Cav-1 levels and decreased cholesterol content in mitochondria, which finally have an effect on slowing down the development of the ALS disease.

In conclusion, progression of ALS is accompanied by a reduction in the Cav-1 protein level, accumulation of cholesterol in crude mitochondria, and decrease in energy metabolism in skeletal muscle. These changes are associated with mitochondrial dysfunction and propagation of oxidative stress. Our data confirm that swim training prolongs the lifespan of ALS mice. Based on our results and the results of other researchers, we can assume that this phenomenon is related to changes in skeletal muscle. Swim training not only improved energy metabolism in skeletal muscle but also, via a Cav-1-related mechanism, maintained mitochondrial function, which resulted in better bioenergetics and lower oxidative stress (Figure 6).

## Supplementary Materials

Supplementary 1 Figure S1: the effects of swim training on lifespan and body weight in the ALS mice. Swim training extends survival (A) in ALS mice, as shown by Kaplan–Meier curves. The mean time to death was delayed by almost 3 weeks (*p* < 0.05) for the swimming ALS mice versus the sedentary ALS mice. Swimming insignificantly delays the reduction of body weight (B). The data are presented as the means ± SEM (*n* = 6 for each group).

Supplementary 2 Figure S2: principal component analysis showing differences in the OXPHOS proteome and glycolysis proteome in the skeletal muscle of the ALS 0, ALS TER, and ALS SWIM mice. (A) 2D graph of variables PC1 and PC2 created with the use of PCA based on the level of 100 identified subunits of mitochondrial respiratory chains measured in ALS mice. (B) 3D graph of variables PC1, PC2, and PC3 created with the use of PCA based on the level of 100 identified subunits of the mitochondrial respiratory chain measured in ALS mice. (C) 2D graph of the variables PC1 and PC2 created with the use of PCA based on the level of 20 identified proteins involved in glycolysis that were measured in ALS mice. The data are presented as the means (squares) and individual results (dots) (*n* = 3 in each group).
