# Supplementary material for: Swim Training Modulates Skeletal Muscle Energy Metabolism, Oxidative Stress, and Mitochondrial Cholesterol Content in Amyotrophic Lateral Sclerosis Mice
Source: Oxid Med Cell Longev. 2018 Apr 11;2018:5940748. doi: 10.1155/2018/5940748 (PMC5924974; doi:10.1155/2018/5940748)
Supplement: Supplementary 1 — Figure S1: the effects of swim training on lifespan and body weight in the ALS mice. Swim training extends survival (A) in ALS mice, as shown by Kaplan–Meier curves. The mean time to death was delayed by almost 3 weeks (p < 0.05) for the swimming ALS mice versus the sedentary ALS mice. Swimming insignificantly delays the reduction of body weight (B). The data are presented as the means ± SEM (n = 6 for each group). [file 5940748.f1.doc]

A.

B
