# Supplementary figures and images for: Swim Training Modulates Skeletal Muscle Energy Metabolism, Oxidative Stress, and Mitochondrial Cholesterol Content in Amyotrophic Lateral Sclerosis Mice
Source: Oxid Med Cell Longev. 2018 Apr 11;2018:5940748. doi: 10.1155/2018/5940748 (PMC5924974; doi:10.1155/2018/5940748)

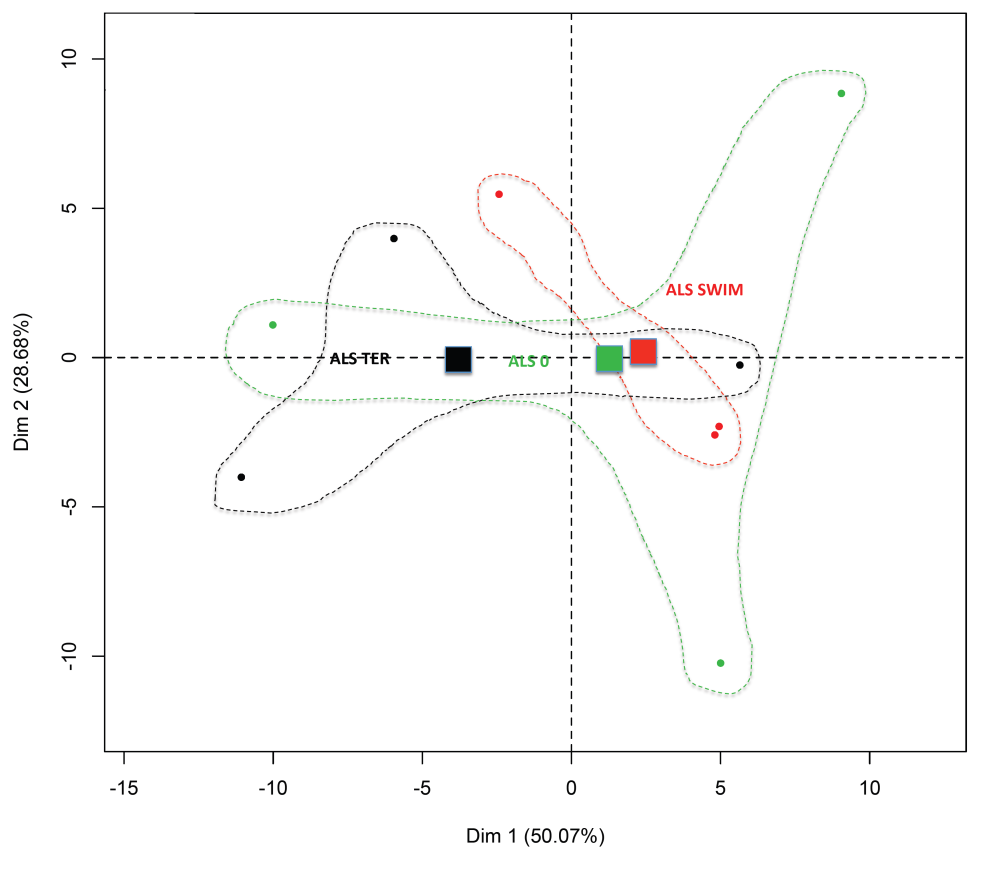


A.


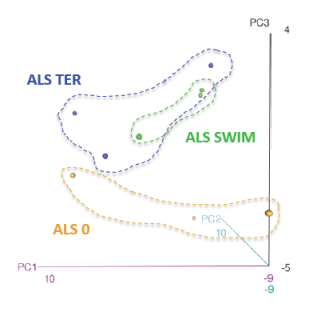

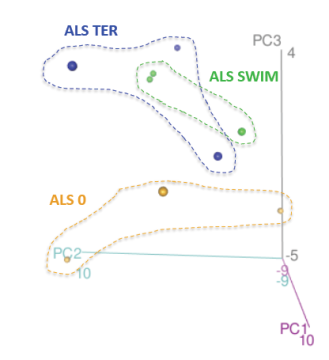

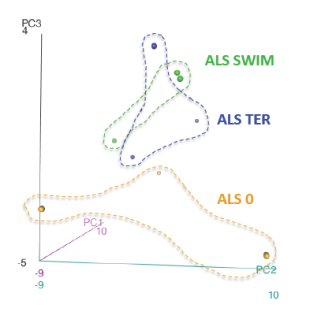


B.


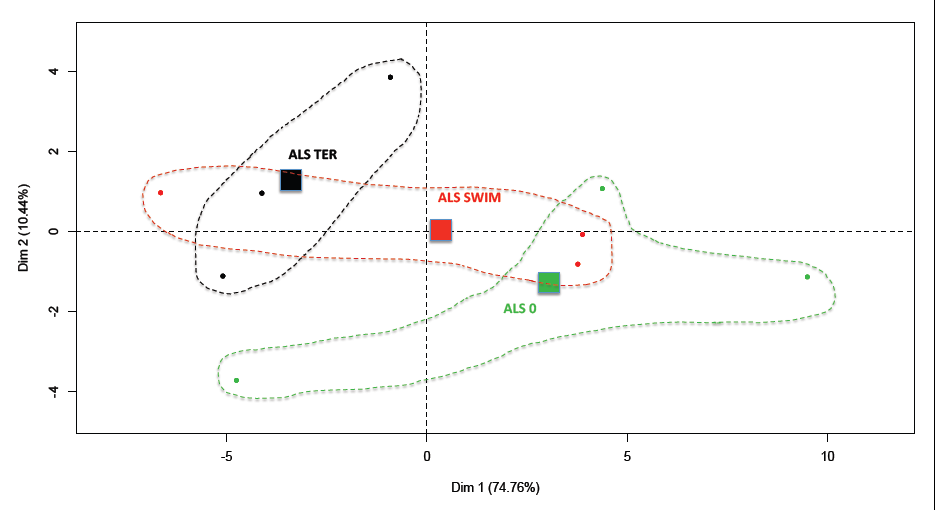


C.


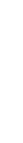

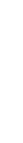

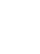

Supplement: Supplementary 2 — Figure S2: principal component analysis showing differences in the OXPHOS proteome and glycolysis proteome in the skeletal muscle of the ALS 0, ALS TER, and ALS SWIM mice. (A) 2D graph of variables PC1 and PC2 created with the use of PCA based on the level of 100 identified subunits of mitochondrial respiratory chains measured in ALS mice. (B) 3D graph of variables PC1, PC2, and PC3 created with the use of PCA based on the level of 100 identified subunits of the mitochondrial respiratory chain measured in ALS mice. (C) 2D graph of the variables PC1 and PC2 created with the use of PCA based on the level of 20 identified proteins involved in glycolysis that were measured in ALS mice. The data are presented as the means (squares) and individual results (dots) (n = 3 in each group). [file 5940748.f2.doc]
